# Supplementary material for: Optical measurement of glutamate release robustly reports short-term plasticity at a fast central synapse
Source: Front Mol Neurosci. 2024 Feb 28;17:1351280. doi: 10.3389/fnmol.2024.1351280 (PMC10933052; doi:10.3389/fnmol.2024.1351280)
Supplement: Supplementary file 1 [file Data_Sheet_1.pdf]

Supplementary Material

1 SUPPLEMENTARY TABLES AND FIGURES

1.1 Figures

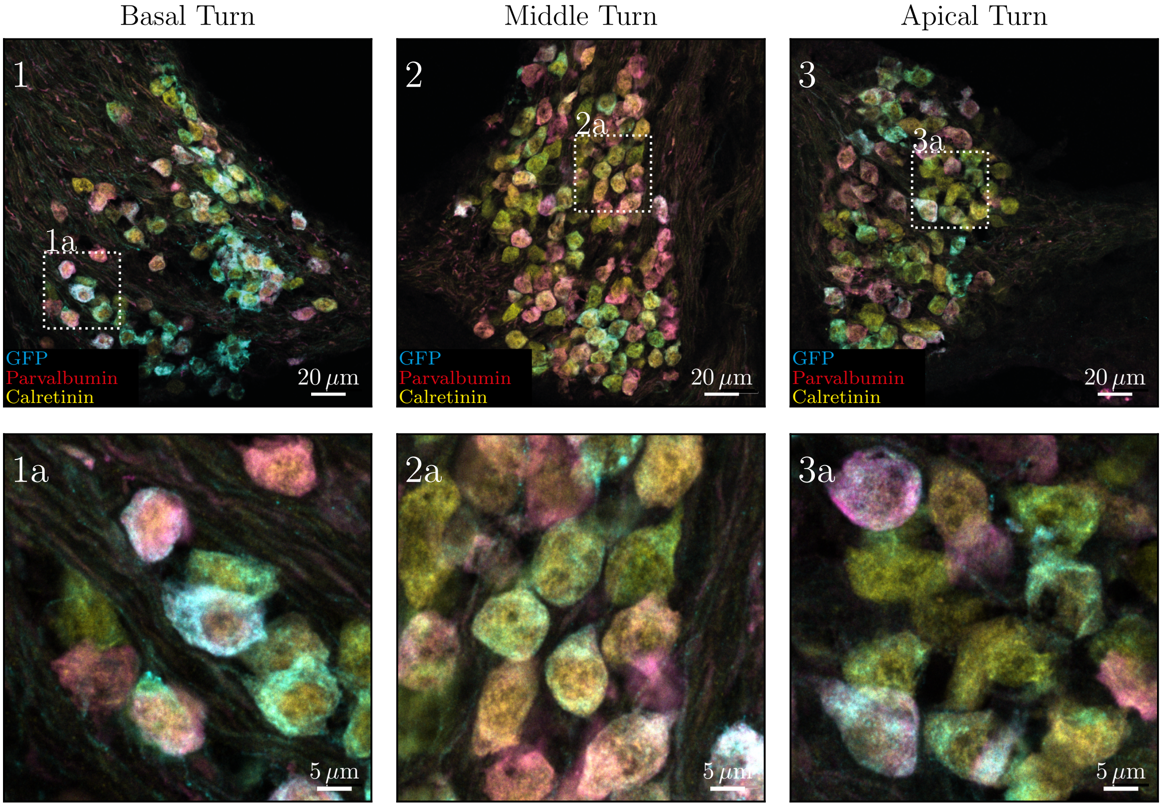

Figure S1. Immunohistochemistry of the cochlea. Confocal sections of cryosections of the spiral ganglion of a mouse cochlea, 11 days after viral transfection. GFP / iGluSnFR were stained using a primary antibody conjugated with Alexa-488, Parvalbumin with a secondary antibody conjugated with Alexa-561 and Calretinin with a secondary antibody conjugated with Alexa-633. In **1**, sections through the basal turn, in **2** trough the middle turn and in **3** through the apical turn of the cochlea are shown. In **1a**, details in $4\times$ zoom are shown. The respective regions are indicated by white squares.

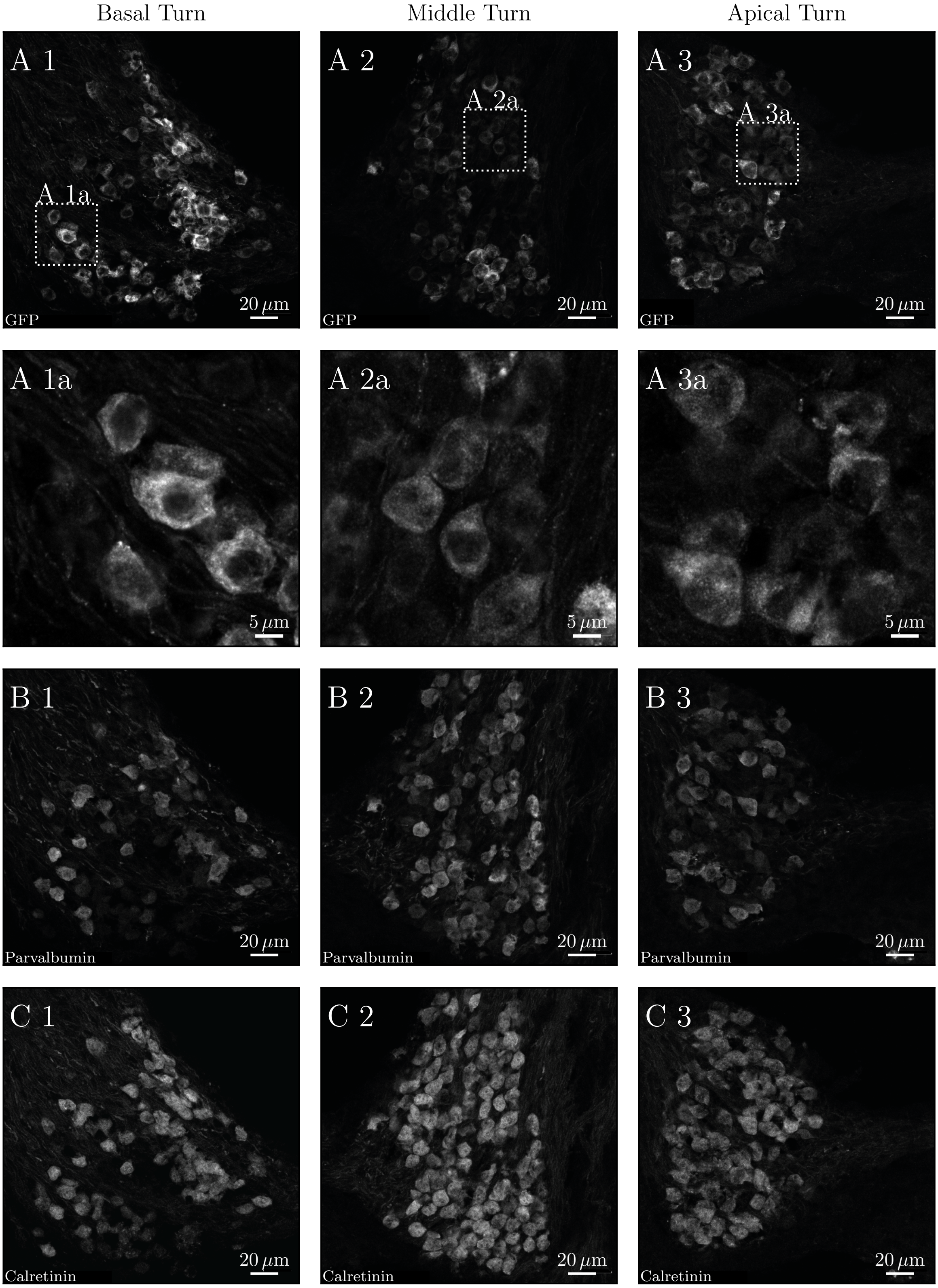

Figure S2. Immunohistochemistry of the cochlea. Grayscale images of the 488 nm / iGluSnFR channel (**A**), the 561 nm / Parvalbumin channel (**B**) and the 633 nm / Calretinin channel (**C**) are shown in the respective panels. For the 488 nm / iGluSnFR channel, in 4 × magnifications, **1a** details are shown in the respective panels. Scale bars in the bottom right corner indicate 20 µm in all sections, except for the magnifications, where they represent 5 µm.

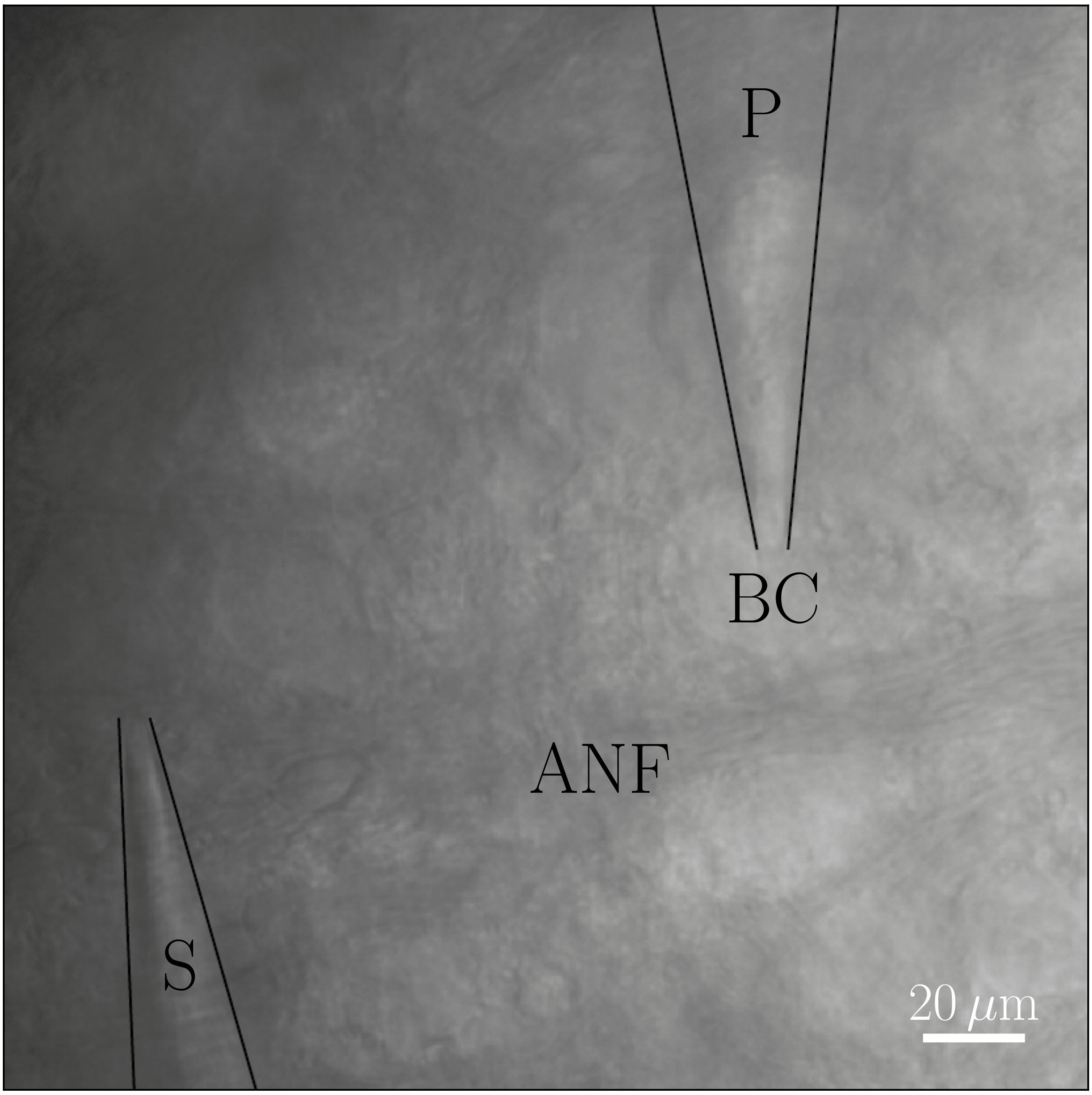

Figure S3. Bright-field example of a typical recording situation. DIC image of the experimental setup in a 150 µm slice of the cochlear nucleus. Here, the conditions during recording are shown. A postsynaptic bushy cell (**BC**) is accessed and voltage-clamped through a patch pipette (**P**), while simultaneously stimulated via a stimulation electrode, located in a saline-filled pipette (tip marked as **S**). In this image, the auditory nerve fibers (**ANF**) which transmit the stimulation signal to the presynaptic axosomatic terminal can be identified. Successful stimulation was, however, not dependent on successful identification of a fiber bundle leading directly to the postsynaptic BC, but was usually achieved by moving the stimulation electrode around the BC, while periodically injecting current in the tissue and monitoring the postsynaptic currents at the same time. Scale bar represents 20 µm.

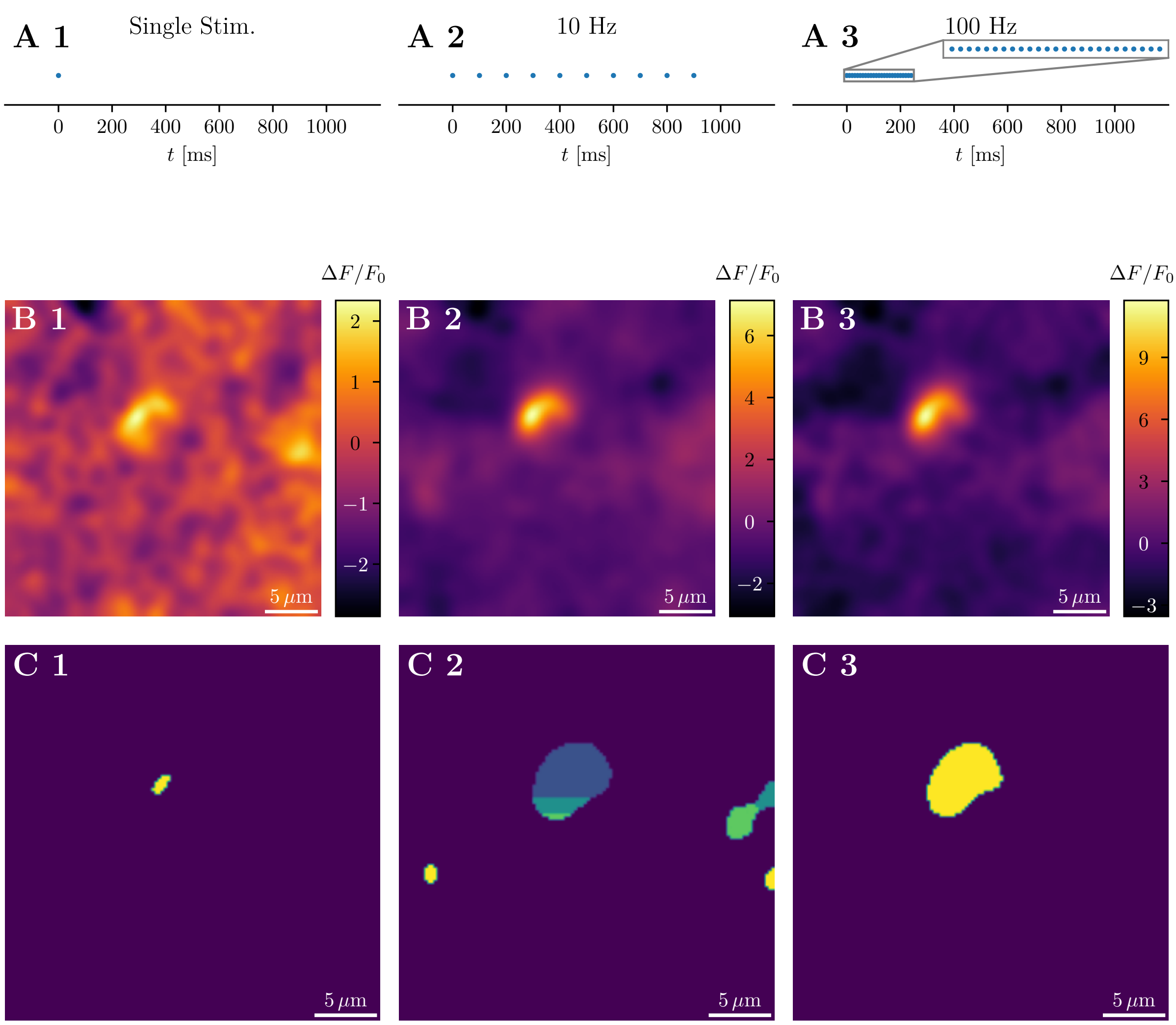

Figure S4. Comparison of ROI identification at different stimulation paradigms. While single stimulation leads to relatively noisy ΔF images, stimulation with 10 stimuli at 10 Hz or 25 stimuli at 100 Hz led to a ΔF image, which resembled terminal morphology more closely and allowed extraction of a meaningful ROI with minimal manual reevaluation. For this figure and further analysis, multiple recordings (usually 3 – 10) for each condition were averaged on a pixel-by-pixel basis. **1**: Single stimulation. Panel **A**: Scheme of stimulation paradigm. Filled dots represent a single afferent fiber stimulation. Panel **B**: ΔF image showing the relative average change in fluorescence of a single pixel from the beginning of the stimulation until $\sim$50 ms after the stimulation. Panel **C**: Automatic ROI detection without manual adjustments. **2**: Low-frequency stimulation of 10 stimuli at 10 Hz. Panel **A**: Scheme of stimulation paradigm in panel **B**: ΔF image showing the relative average change from the beginning of the stimulation until $\sim$1000 ms after the stimulation. Panel **C**: Automatic ROI detection without manual adjustments. **3**: High-frequency stimulation with 25 stimuli at 100 Hz. Panel **A**: Scheme of stimulation paradigm (25 stimuli at 100 Hz, magnified in inset to show individual stimulations). Panel **B**: ΔF image showing the relative average change from the beginning of the stimulation until $\sim$390 ms after the stimulation. Panel **C**: Automatic ROI detection without manual adjustments.
Small detected segments occurring outside of the central, labeled segment were removed manually afterwards, if they occurred, as they most likely either represent imaging noise or terminals projecting onto neighboring cells. Scale bars represent 5 μm.

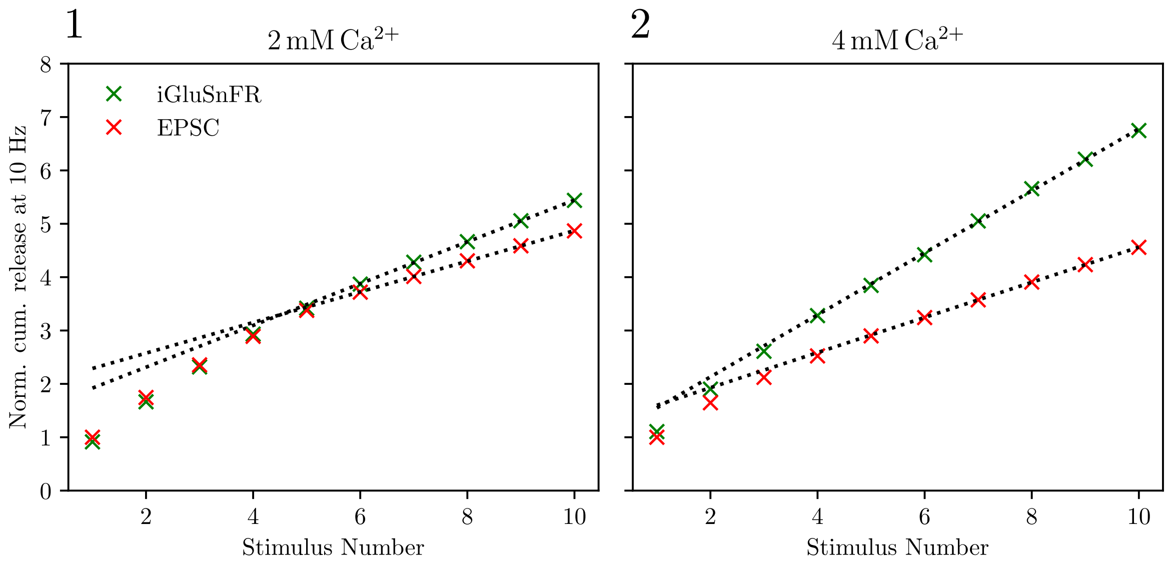

Figure S5. Cumulative release analysis of 10 Hz data. Cumulative release was derived by summation of peak amplitudes of consecutive events in electrophysiological data and deconvolved iGluSnFR signal (in this case of the 10 Hz data). Cumulative release was normalized by dividing by the single response at the respective $[Ca^{2+}]_e$ levels. Panel **1** show the normalized average cumulative release, derived from measurements of $N = 7$ animals, $n = 8$ cells, when stimulated with 5 or 10 stimuli at 10 Hz at 2 mM $[Ca^{2+}]_e$. Panels **2** show the average normalized cumulative release from $N = 7$ animals, $n = 9$ cells, when stimulated with 10 stimuli at 10 Hz at 4 mM $[Ca^{2+}]_e$.

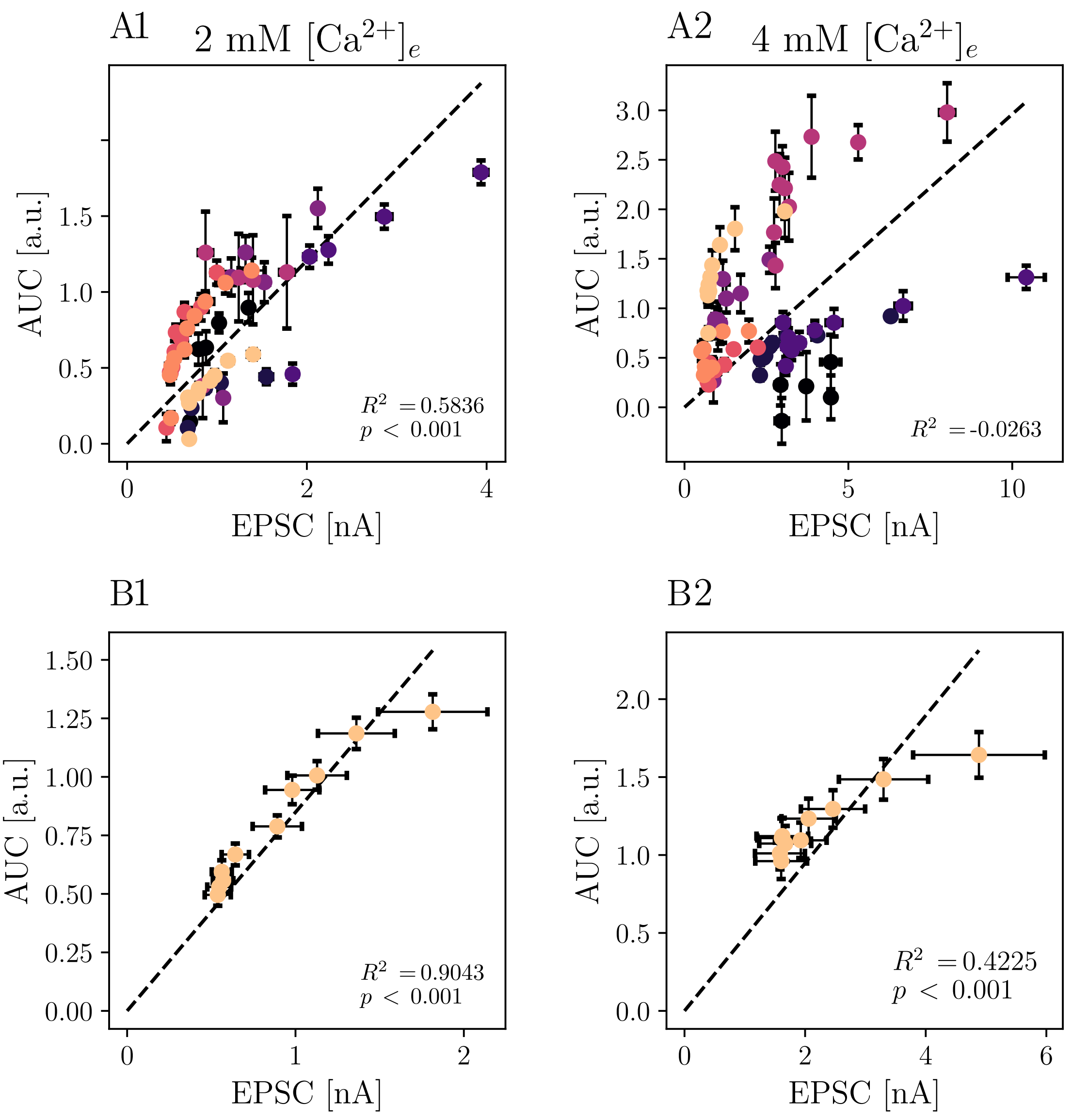

Figure S6. Non-normalized data of iGluSnFR vs. eEPSC data. Plots in **A** show the absolute amplitudes of AUC and eEPSC of the same data presented in fig. 6, **A**. Plots in **B** show the absolute amplitudes of AUC and eEPSC of the same data presented in fig. 6, **B**. The non-normalized data for 4 mM $[Ca^{2+}]_e$ was not fit well by a single linear function, likely due to larger variance between cells.

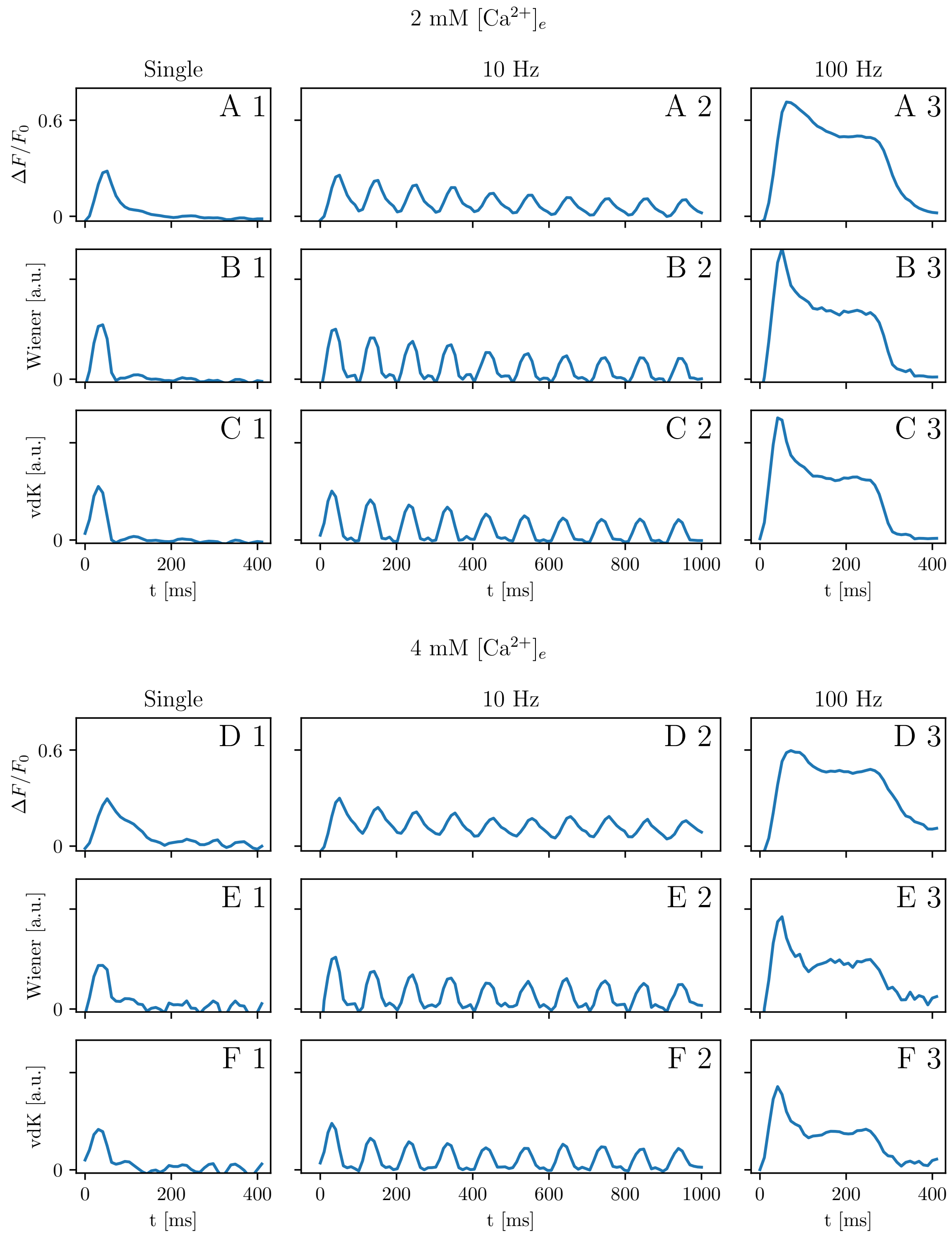

Figure S7. Deconvolved traces of averaged recordings. Recordings of $N = 7$ animals, $n = 8$ cells at 2 mM $[Ca^{2+}]_e$ (panels **A – C**) and $N = 7$ animals, $n = 9$ cells at 4 mM $[Ca^{2+}]_e$ (panels **D – F**) were averaged and filtered (panels **A / D**). Either a Wiener deconvolution algorithm (panels **B / E**, ”Wiener”) or a deconvolution algorithm based on Cohen et al. (1981) (panels **C / F**, ”vdK”) was applied to the traces with single stimuli (panels **1**), 10 Hz traces (panels **2**) or 100 Hz traces (panels **3**). Both methods have been used (in variations) in the analysis of electrophysiological (Cohen et al., 1981; Van der Kloot, 1988), as well as glutamate imaging data (Taschenberger et al., 2016; Sakamoto et al., 2018). They both share the assumptions that (a) the iGluSnFR signal rises instantaneously, (b) glutamate release occurs completely synchronized and (c) iGluSnFR responses are added linearly and are indifferent to the history of responses. At our hands, results with both methods were roughly comparable. For further analysis, the analytical solution was used, as it has been proven to be more reliable for deconvolving electrophysiological responses (Van der Kloot, 1988).

REFERENCES

Cohen, I., Van Der Kloot, W., and Attwell, D. (1981). The timing of channel opening during miniature end-plate currents. *Brain Research* 223, 185–189. doi:10.1016/0006-8993(81)90821-0

Sakamoto, H., Ariyoshi, T., Kimpara, N., Sugao, K., Taiko, I., Takikawa, K., et al. (2018). Synaptic weight set by Munc13-1 supramolecular assemblies. *Nature Neuroscience* 21, 41–49. doi:10.1038/s41593-017-0041-9

Taschenberger, H., Woehler, A., and Neher, E. (2016). Superpriming of synaptic vesicles as a common basis for intersynapse variability and modulation of synaptic strength. *Proceedings of the National Academy of Sciences* 113, E4548–E4557. doi:10.1073/pnas.1606383113

Van der Kloot, W. (1988). Estimating the timing of quantal releases during end-plate currents at the frog neuromuscular junction. *The Journal of Physiology* 402, 595–603. doi:10.1113/jphysiol.1988.sp017224
